# Constitutively Active Canonical NF-κB Pathway Induces Severe Bone Loss in Mice

**DOI:** 10.1371/journal.pone.0038694

**Published:** 2012-06-07

**Authors:** Jesse E. Otero, Tim Chen, Kaihua Zhang, Yousef Abu-Amer

**Affiliations:** 1 Department of Orthopedic Surgery, University of Iowa Hospitals and Clinics, Iowa City, Iowa, United States of America; 2 Department of Orthopedic Surgery and Cell Biology and Physiology, Washington University School of Medicine, Saint Louis, Missouri, United States of America; Ohio State University, United States of America

## Abstract

Physiologic osteoclastogenesis entails activation of multiple signal transduction pathways distal to the cell membrane receptor RANK. However, atypical osteoclastogenesis driven by pro-inflammatory stimuli has been described. We have reported recently a novel mechanism whereby endogenous mutational activation of the classical NF-κB pathway is sufficient to induce RANKL/RANK-independent osteoclastogenesis. Here we investigate the physiologic relevance of this phenomenon *in vivo*. Using a knock-in approach, the active form of IKK2, namely IKK2SSEE, was introduced into the myeloid lineage with the aid of CD11b-cre mice. Phenotypic assessment revealed that expression of IKK2SSEE in the myeloid compartment induced significant bone loss *in vivo*. This observation was supported by a dramatic increase in the number and size of osteoclasts in trabecular regions, elevated levels of circulating TRACP-5b, and reduced bone volume. Mechanistically, we observed that IKK2SSEE induced high expression of not only p65 but also p52 and RelB; the latter two molecules are considered exclusive members of the alternative NF-κB pathway. Intriguingly, RelB and P52 were both required to mediate the osteoclastogenic effect of IKK2SSEE and co-expression of these two proteins was sufficient to recapitulate osteoclastogenesis in the absence of RANKL or IKK2SSEE. Furthermore, we found that NF-κB2/p100 is a potent inhibitor of IKK2SSEE-induced osteoclastogenesis. Deletion of p52 enabled more robust osteoclast formation by the active kinase. In summary, molecular activation of IKK2 may play a role in conditions of pathologic bone destruction, which may be refractory to therapeutic interventions targeting the proximal RANKL/RANK signal.

## Introduction

Bone metabolism is tightly regulated by the actions of osteoblasts and osteoclasts throughout life [1]. Whereas osteoblasts are characterized as bone forming cells laying down organic matrix, the primary function of osteoclasts is resorbing and remodeling bones. Under physiologic conditions a balance regulating bone formation and bone resorption is maintained. Specifically, mesenchymal cells secrete factors such as RANK ligand and osteoprotegerin to maintain and regulate osteoclast formation and survival. However, under pathologic conditions the delicate balance between bone formation and bone resorption is disrupted contributing to many bone disorders [2], [3].

Osteoclast differentiation entails binding of RANK ligand to its cognate receptor on myeloid progenitor cells and subsequent activation of multiple intracellular pathways including AKT/PI3K, MAP kinase, and NF-κB [4]. The critical role of NF-κB in osteoclast differentiation and bone homeostasis has been widely investigated [5], [6]. This ubiquitous pathway controls physiologic and pathologic responses in various tissues and encompasses several critical kinases and signaling molecules with varying degrees of contribution to tissue health and disease [7], [8]. These proteins have been classified into canonical and non-canonical NF-κB pathways. The former pathway which is activated by RANK ligand as well as by inflammatory stimuli such as TNF and IL-1β, is regulated by the IKK complex dominated by IKK2 and IKKγ/NEMO leading to phosphorylation of IκB and activation of p50, p65, cRel transcriptional complexes. On the other hand, the non-canonical NF-κB pathway is regulated by NF-κB-inducing kinase (NIK) which in turn activate IKK1 leading to proteolytic processing of the inhibitory protein p100 and releasing p52 and RelB, the downstream effectors of this pathway [9]. Genetic studies have shown that all members of canonical and non-canonical NF-κB pathway are crucial for normal skeletal development and bone health. In this regard, deletion of IKK1 [10], IKK2 [11], [12], NEMO (unpublished data and [13]), NIK, p52/p50, p65, RelB, and cRel resulted with impaired osteoclast differentiation and/or function and abnormal skeletal development [14]–[16].

Despite the exclusive role of RANK ligand as the master regulator of physiologic osteoclastogenesis and bone homeostasis, alternative pathways distinct from RANK ligand signaling affecting osteoclastogenesis and bone metabolism have been described. These include primarily pathologic stimulators, chiefly TNF. In this regard, it has been demonstrated that aside from its RANKL-costimulatory osteoclastogenic function, TNF induces osteoclast formation in RANK/RANKL-deficient mice, in vivo, albeit in the absence of NF-κBp100 subunit [17]. More recently, we have demonstrated that an IKK2 gain of function mutation (IKK2 in which serines 177/181 were substituted with glutamates; namely IKK2SSEE) induces osteoclastogenesis independent of upstream RANK/RANKL and TNF/TNFr signaling [18]. This constitutively active IKK2-dependent osteoclastogenesis was also independent of IKK1, and NEMO. In this study, we show that expression of IKK2SSEE in the myeloid compartment induces significant bone loss in vivo. This observation is supported by a significant increase in the number and size of osteoclasts in trabecular regions, elevated levels of circulating TRACP-5b, and reduced bone volume. Mechanistically, we report the novel finding that IKK2SSEE activates RelB and p52, two components of the non-canonical NF-κB pathway. More importantly, RelB and P52 are both required to mediate the osteoclastogenic effect of IKK2SSEE and co-expression of these two proteins is sufficient to recapitulate osteoclastogenesis.

## Results

### IKK2SSEE transgenic mice (IKK2SSEE^MYELO^) display severe bone loss

**Figure 1:**
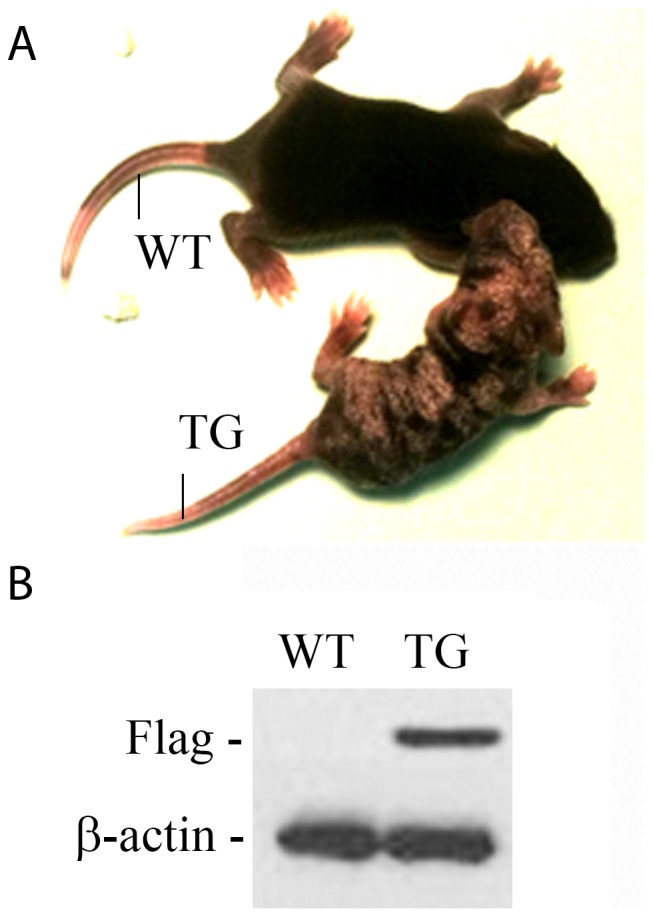
Generation of IKK2SSEE^MYELO^ -tg mice. We utilized the R26StopIkk2SSEE mice in which a cDNA encoding IKK2 containing two serine to glutamate substitutions in the activation loop of the kinase domain, preceded by a loxP-flanked STOP cassette, was cloned into the ubiquitously expressed ROSA26 locus. We crossed mice carrying this allele with the CD11b-cre mice in order to express IKK2SSEE exclusively in myeloid cells, including osteoclasts and osteoclast precursors (IKK2SSEE^MYELO^ –tg). A) Photograph of 2-week old wild type (WT) and transgenic (TG) mice. B) Western blot detection of Flag-IKK2SEE (using anti-Flag antibody) from corresponding bone marrow lysates.

**Figure 2:**
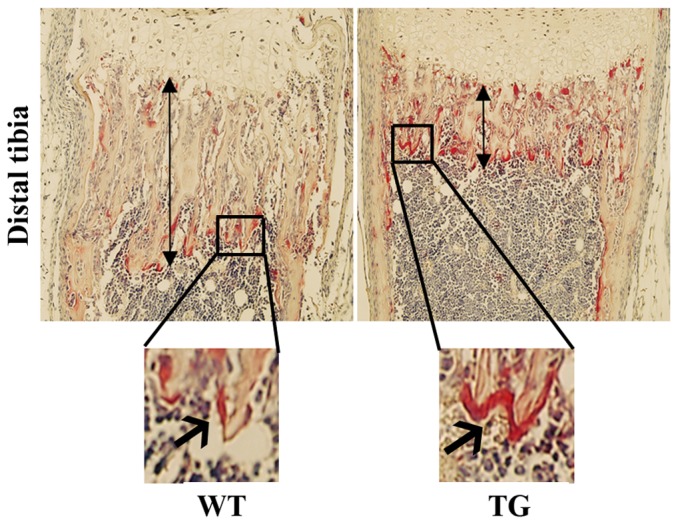
Decreased trabecular bone volume and increased osteoclasts in IKK2SSEE^MYELO^ -tg mice. Tibia from wild type (WT) and transgenic mice (TG) were histologically processed and stained with tartrate-resistant acid phosphatase to detect osteoclasts. Top panels display the growth plate and the proximal diaphysis regions. Double-headed arrows indicate the trabecular region. Insert is magnification (40×) of trabecular region from WT and TG sections showing giant osteoclasts (red) engulfing trabeculi. Images are representative of five different experiments.

**Figure 3:**
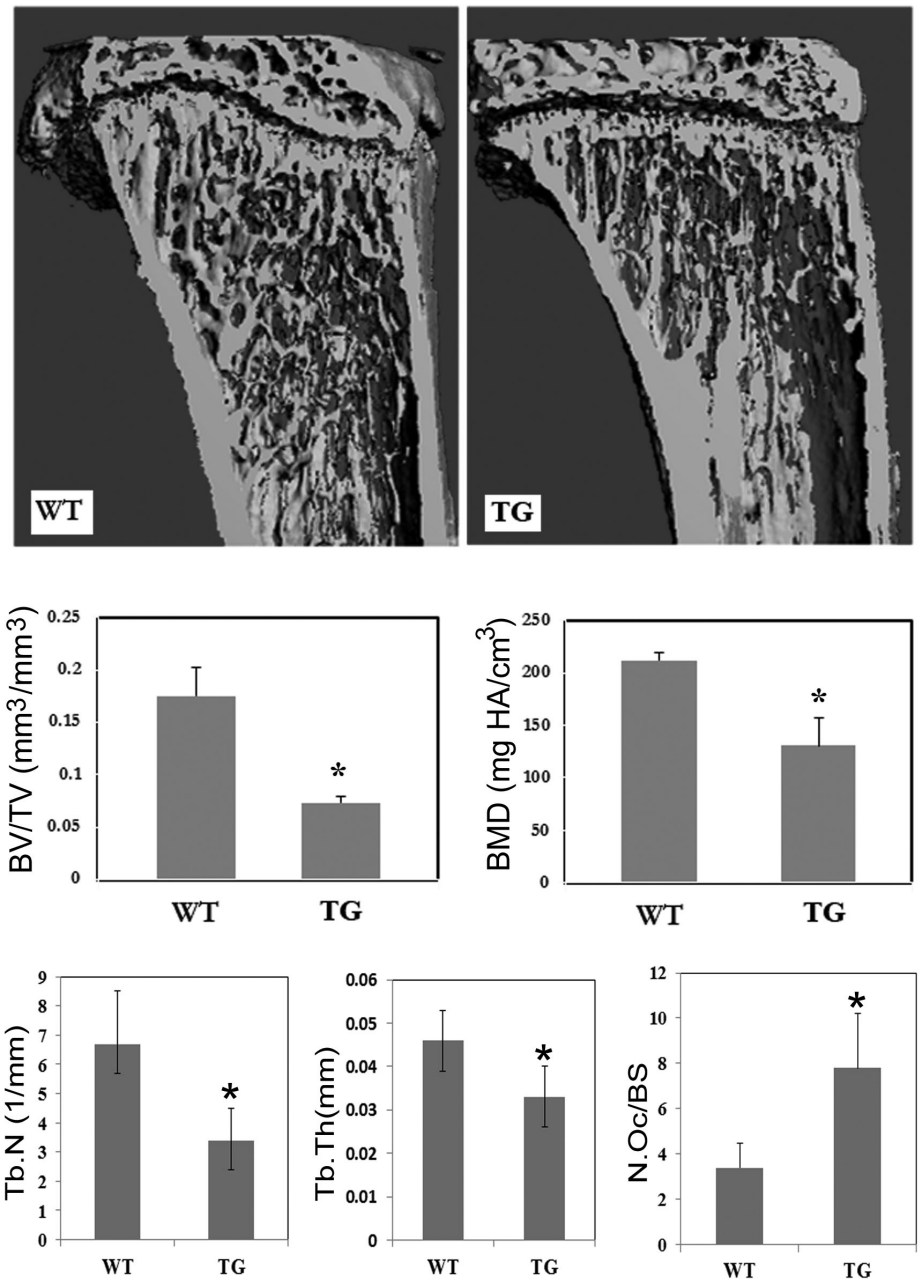
Bone mineral density is reduced in IKK2SSEE^MYELO^ -tg mice. Long bones of 4-week old wild type (9 mice) and transgenic (8) mice were processed and analyzed by micro CT scanning as described under methods. Absolute values of bone mineral density (BMD), trabecular number (Tb.N), trabeculat thickness (Tb.Th), number of osteoclasts per bone surface (N.Oc/BS) and bone volume/total volume (BV/TV) between WT and TG mice are depicted. *P<0.05.

**Figure 4:**
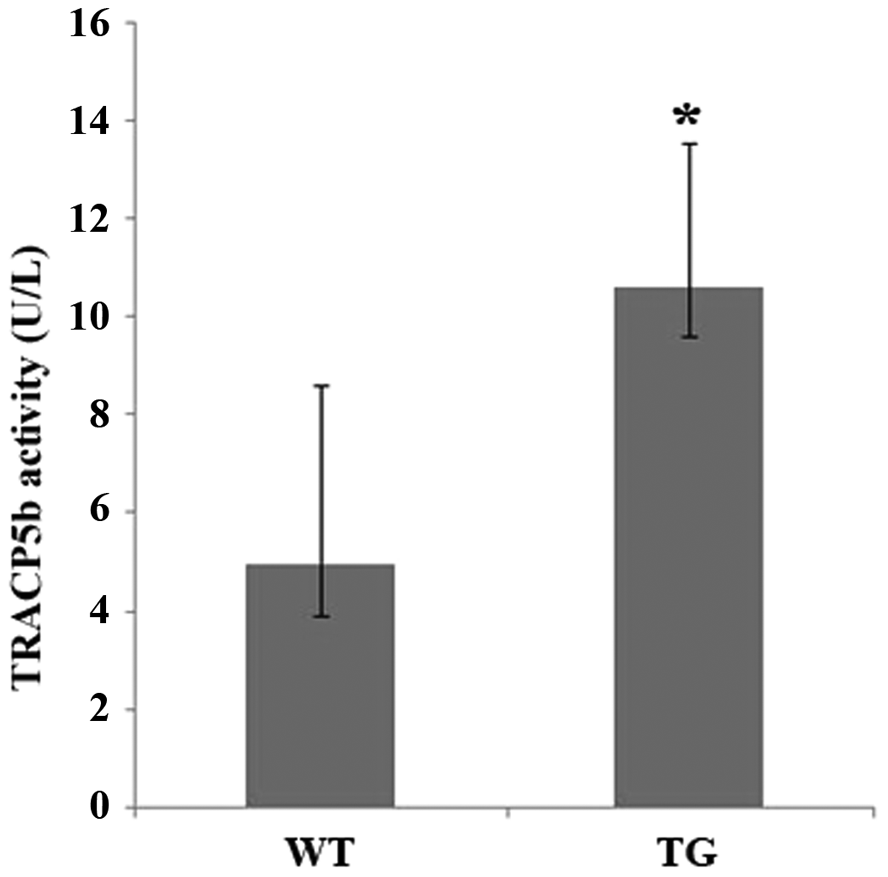
Serum levels of TRACP5b are elevated in IKK2SSEE^MYELO^ -tg mice. Serum was collected from wild type (9) and transgenic (8) mice and levels of the enzyme TRACP5b (U/L +/−SE) were measured. * denotes p<0.05.

We have shown previously that the active form of IKK2, IKK2SSEE, induces RANKL-independent osteoclastogenesis, in vitro [18]. To determine the physiological significance of this finding, we examined its role in vivo through expression of constitutively active IKK2 in a tissue-specific manner. Specifically, we employed the R26StopIKK2SSEE mice in which a cDNA encoding IKK2 containing two serine to glutamate substitutions in the activation loop of the kinase domain, preceded by a loxP-flanked STOP cassette, was cloned into the ubiquitously expressed ROSA26 locus [19]. We crossed mice carrying this allele to the CD11b-cre mice in order to express IKK2SSEE exclusively in myeloid cells, inclusive of osteoclast progenitors. Mice were born and survived an average of 6 weeks of age. The IKK2SSEE transgenic mice (herein referred to as IKK2SSEE^MYELO^) exhibited growth retardation and severe skin inflammation (fig. 1A). Both macrophages and osteoclasts derived from IKK2SSEE^MYELO^ express Flag-tagged IKK2SSEE detected by western blot (fig. 1B). Histological (fig. 2), micro-CT and histomorphometric (fig. 3) analyses showed that IKK2SSEE^MYELO^ transgenic mice have significantly less trabecular bone compared with the wild type (WT) mice, while no significant reduction of cortical bone was observed (not shown). Specifically, bone mineral density (BMD) in transgenic mice was 58.9% of that in WT mice. BV/TV values were 0.177 mm3/mm3 in WT compared to 0.068 mm3/mm3 in transgenic mice. Closer examination revealed that trabecular bone sections of IKK2SSEE transgenic mice contain more and larger osteoclasts attached to trabeculi (fig. 2, insert). Indeed, histomorphometric quantification using Osteomeasure (Osteometrics, Inc) revealed that the trabecular region of transgenic mice contains nearly twice as much osteoclasts compared with WT mice (fig. 3). Supporting a functional significance of this observation, IKK2SSEE^MYELO^ transgenic mice have two-fold increase of serum TRACP 5b activity (fig. 4) (10.59 U/L vs 4.93 U/L), reflecting an overall heightened bone resorptive activity of osteoclasts in these mice.

#### Bone marrow macrophages derived from IKK2SSEE transgenic mice undergo spontaneous differentiation into osteoclasts

**Figure 5:**
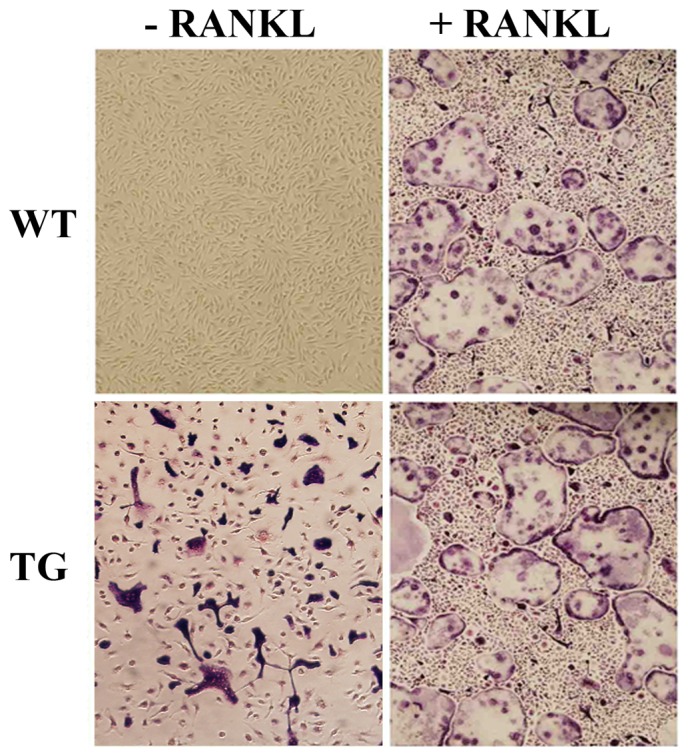
Bone marrow macrophages derived from IKK2SSEE^MYELO^ -tg mice generate RANKL-independent osteoclasts. Marrow macrophages were isolated from wild type and IKK2SSEE^MYELO^ -tg mice and cultured with M-CSF in the absence or presence of RANKL (50 ng/ml). Cultures were fixed on day 5 of culture and stained with TRAP. Purple/red cells are TRAP-positive *bona fide* osteoclasts which are capable of resorbing bone (data not shown). Number of osteoclasts per well (Average of 4 wells from 5 different experiments (+/−SE): WT+RANKL 215+/−32; TG 105+/−17; TG+RANKL 248+/−41.

**Figure 6:**
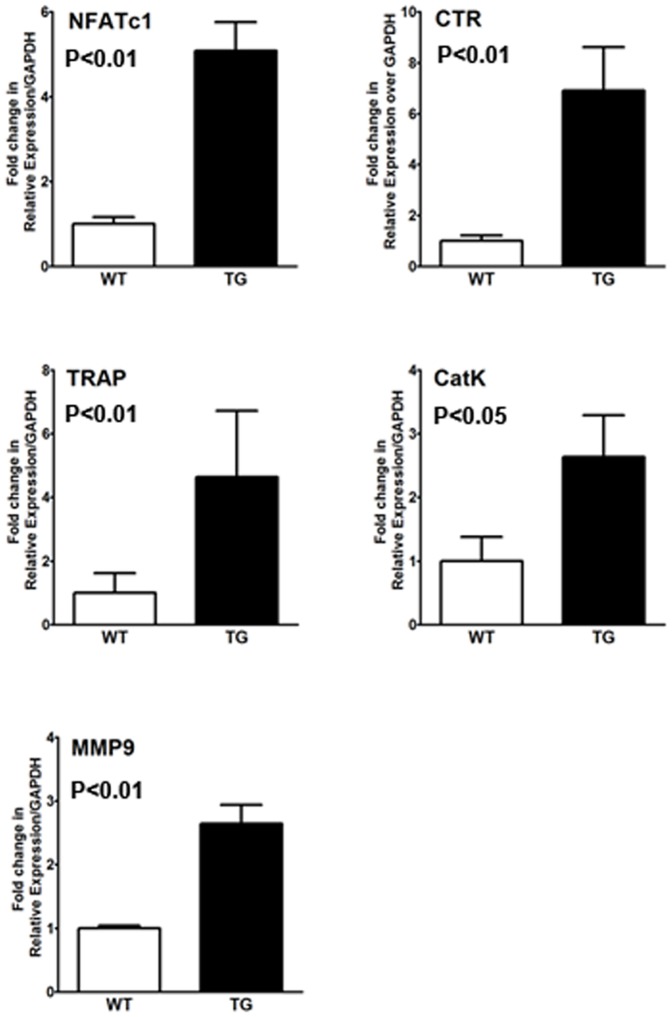
Markers of osteoclast are increased in cells derived from IKK2SSEE^MYELO^ -tg mice. Bone marrow macrophages were isolated from WT and IKK2SSEE-tg mice and cultured in the presence of M-CSF for 4 days. RNA was then extracted and subjected to real-time Q-PCR to detect expression of the indicated markers. p<0.01 for NFATc1, p<0.01for TRAP, p<0.05 for Cathepsin K, p<0.01 for MMP9, and p<0.01 for calcitonin receptor (CTR).

To determine the osteoclastogenic potential of IKK2SSEE transgenic bone marrow macrophages, these cells were cultured in vitro with M-CSF alone. Consistent with its intrinsic osteoclast effect in vitro, IKK2SSEE-expressing bone marrow macrophages isolated from these transgenic mice were capable of forming osteoclasts ex-vivo in the absence of RANKL (fig. 5). Furthermore, real-time PCR showed that IKK2SSEE upregulated NFATc1 expression in macrophage/osteoclast lineage (fig. 6). IKK2SSEE also significantly enhanced expression of major osteoclast markers including TRAP, Cathepsin K, MMP9 and calcitonin receptor expression compared to the wild type control (fig. 6).

### Expression and secretion of osteoclastogenic factors (RANKL and OPG) and inflammatory factors in IKK2SSEE^MYELO^ mice

**Figure 7:**
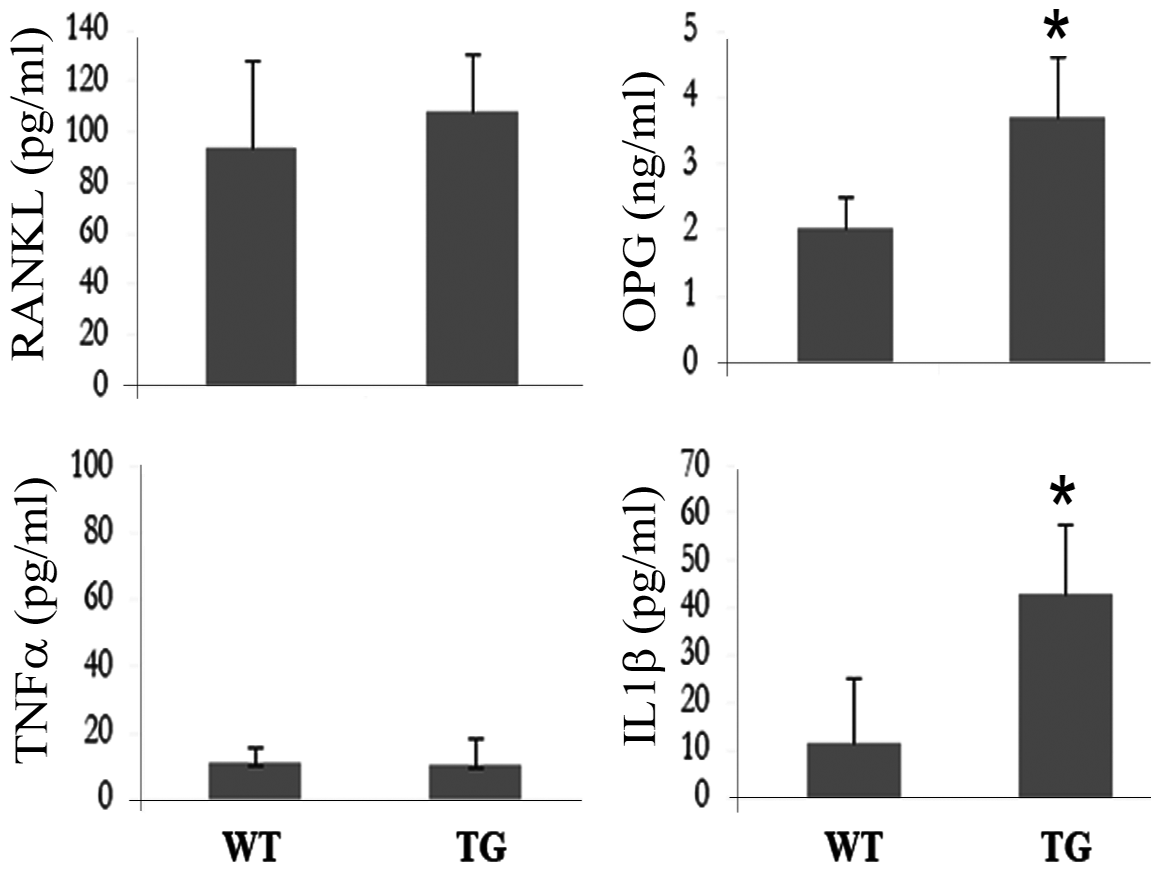
Expression levels of IL-1β and OPG are higher in the serum of IKK2SSEE^MYELO^ -tg mice. IL-1β, OPG, RANKL and TNFα levels in the serum of wild type and transgenic mice were measured using commercial ELISA kits. * denotes p<0.05 (IL-1β) and p<0.05 (OPG).

To explore the mechanism underlying enhanced osteoclast activity in IKK2SSEE^MYELO^-tg mice, we measured circulating levels of osteoclastogenic factors (RANKL and OPG) and inflammatory factors (TNFα and IL1β) which function cooperatively with RANKL. Serum level of RANKL and TNFα remain normal, however serum levels of IL1β increased significantly compared to wild type counterparts (fig. 7). Interestingly, serum level of OPG also increased dramatically, probably reflecting compensatory role of OPG in IKK2SSEE^MYELO^ -tg mice. To determine if elevated levels of IL-1β are responsible for the IKK2SSEE osteoclastogenic effect, IKK2SSEE transgenic mice were cross bred with IL-1r knockout mice. However, all parameters of osteoclastogenesis examined in these hybrid mice were similar to those derived from the IKK2SSEE^MYELO^-tg mice (not shown).

#### IKK2SSEE induces osteoclastogenesis via activation of NF-κB alternative pathway

**Figure 8:**
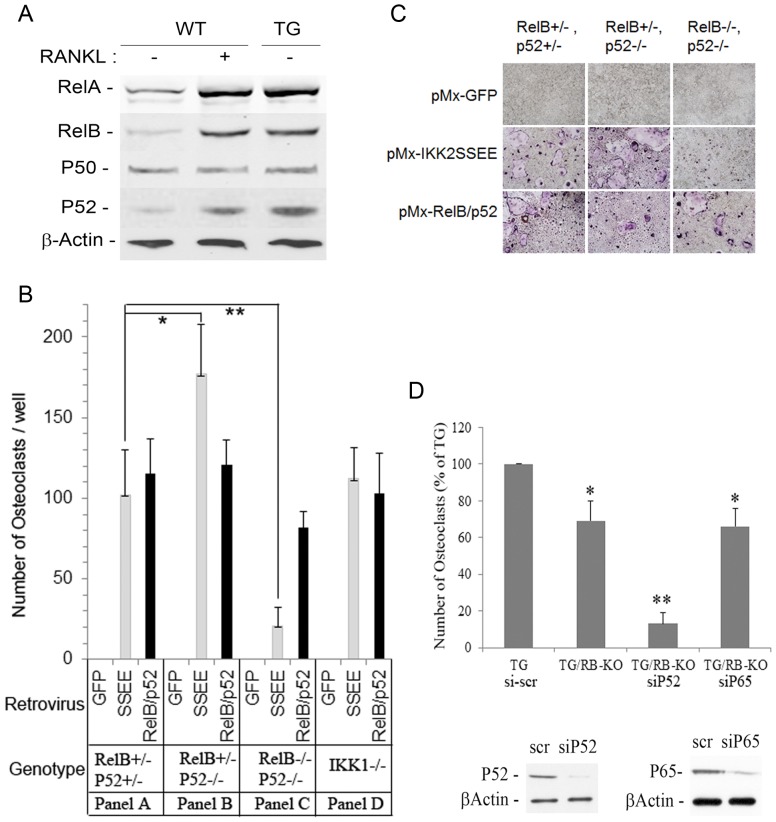
NF-κB subunits RelB and p52 mediate the osteoclastogenic effect of IKK2SSEE. A) Marrow macrophages were isolated from WT and IKK2SSEE transgenic (TG) mice and plated in culture for 5 days in the presence of M-CSF. One set of WT cells was treated with RANKL (to serve as a positive control). Total cell lysates were subjected to Western blots as indicated. B) Marrow macrophages were isolated from mice with the indicated gene deletions (denoted by genotype at the lower part of the figure). Cells were infected with retrovirus expressing the indicated gene products (denoted by retrovirus) and were cultured with M-CSF in the absence of RANKL. Cell cultures were fixed and TRAP-stained on day 5. Multi-nucleated (≥3 nuclei/cells) TRAP-positive cells were counted from three different wells for each condition. The data represent the average of three independent experiments. *p<0.01, **p<0.05. C) Representative images of osteoclast cultures depicted in fig. 8B. D) IKK2SSEE transgenic mice alone or crossed with RelB-KO mice (hybrid denoted TG/RB-KO) were used. Osteoclasts were generated (in the absence of RANKL) from marrow macrophages isolated from IKK2SSEE-transgenic mice and from hybrid TG/RB-KO mice. In addition, osteoclasts were generated from marrow macrophages isolated from the latter mouse in which either P52-NF-κB or RelA were silenced using siRNA approach. Bottom panels of 8C are western blots for p52 and p65 (RelA) from control (scr = scramble) and silenced (si) conditions. Knockdown efficiency exceeded 80% of control.

We have shown previously that IKK2SSEE induces expression of RelA/p65 (canonical pathway) as well as RelB and p52 (non-canonical pathway) in IKK1-null cells [18]. Consistent with these findings, we now show that expression of these NF-κB subunits is also elevated in cells derived from IKK2SSEE transgenic mice (Fig. 8A), suggesting that one or a combination of these subunits maybe responsible for the osteoclastogenic effect of IKK2SSEE. Thus, we surmised that expression of these NF-κB subunits at various combinations may recapitulate the IKK2SSEE osteoclastogenic effect. To this end, expression of RelA, RelB, or p52 individually did not alter the RANKL-independent osteoclastogenic potential of the cells, as was the case when RelA and RelB were co-expressed (not shown). Surprisingly, however, co-expression of p52 and RelB, both of which are components of the non-canonical NF-κB activation pathway (normally induced by IKK1), elicited RANKL-independent osteoclastogenesis, in vitro (fig. 8B, panel A, and corresponding images in fig. 8C). To determine if indeed p52 and RelB subunits are essential mediators of this osteoclastogenic process, we used P52-KO and p52/RelB double knockout cells. The results depicted in figure 8B (panels B–C) show that whereas IKK2SSEE induced robust osteoclastogenesis in p52 null cells expressing RelB (fig. 8B, panel B), suggesting that NF-κB2/p100 is an endogenous inhibitor of IKK2SSEE, combined deletion of p52NF-κB and RelB abrogated the IKK2SSEE osteoclastogenic effect (gray bar). To further verify the specificity of this event, the osteoclastogenic effect of IKK2SSEE was examined using bone marrow macrophages isolated from IKK1-null cells. Supporting a specific IKK2SSEE effect, combined expression of p52 and RelB NF-κB subunits in IKK1-null cells mimicked IKK2SSEE-induced osteoclastogenesis (fig. 8B, panel D). Taken together, the data provides strong correlation between IKK2SSEE and P52/RelB-induced osteoclastogenesis. To further determine if this evidence holds true in the transgenic mouse model, IKK2SSEE-TG mouse was crossed with the RelB germ line knockout mouse. Marrow macrophages derived from the hybrid mouse, termed TG/RB-KO, were subjected to siRNA knockdown of either P52 or P65/RelA NF-κB subunits (siP52, siP65). Control conditions were treated with scrambled sequence (si-scr) or vehicle. Cells were then cultured in the presence of M-CSF, and osteoclasts were determined in 6 day old culture using TRAP staining assay. The results summarized in figure 8D indicate that whereas deletion of RelB reduced osteoclasts by approximately 31%, combined elimination of P52 (by siRNA knockdown) and RelB (by genetic deletion) significantly abrogated the IKK2SSEE-TG osteoclastogenic effect (∼87% reduction compared with TG control). On the other hand, knockdown of P65 subunit in the RelB-KO background had little effect on IKK2SSEE-TG-induced osteoclastogenesis. Altogether, our findings support the notion that p52 and RelB are the primary mediators of the IKK2SSEE osteoclastogenic effect in vitro and in vivo.

## Discussion

We published previously that the constitutively active form of IKK2, namely IKK2SSEE, induces RANKL-independent osteoclastogenesis [18]. We also have established that deletion of the IKK2 gene in the myeloid compartment impedes osteoclastogenesis [11]. In the current study, we examined the endogenous osteoclastogenic role of IKK2SSEE. Restricted myeloid expression of the IKK2SSEE transgene led to severe bone loss primarily in the trabecular compartment. Further examination revealed an increased number and size of osteoclasts corresponding with elevated serum levels of TRACP5b in IKK2SSEE transgenic mice compared with control counterparts. These results suggest that activating mutations in the IKK2SSEE are sufficient to induce an osteolytic disease.

Further support for the cell-autonomous intrinsic osteoclastogenic effect of IKK2SSEE stems from our observation that this phenomenon appears to be independent of the osteoclastogenic and inflammatory factors TNFα and IL1β. Although levels of the latter cytokine were elevated under IKK2SSEE conditions, deletion of IL1R and TNFr1 failed to alter the osteoclastogenic effect of IKK2SSEE, suggesting that TNF and IL-1β are dispensable for this phenomenon, and thus are presumed non-essential mediators of this process. These findings are further supported by our observation that retroviral transduction of IKK2SSEE into IL1 receptor deficient monocytes induces RANKL-independent osteoclastogenesis in vitro (data not shown). Altogether, these findings document uncoupling of the IKK2SSEE-induced inflammatory and osteoclastogenic events. However, the functional significance of this finding remains to be investigated.

The most intriguing finding of our study was induction of RelB and p52 expression by IKK2SSEE, two NF-κB subunits traditionally induced by IKK1 and considered exclusive mediators of the non-canonical NF-κB pathway. Consistent with this result, co-expression of RelB and p52 subunits, but not individually, induced osteoclastogenesis independent of RANKL and IKK1. This observation suggests that these two non-canonical NF-κB subunits concomitantly mediate the osteoclastogenic effect of IKK2SSEE. The finding that IKK2SSEE and RelB/p52-induced osteoclastogenesis ensues in the absence of the RANKL cytokine and the IKK1gene presents a potential novel mechanism whereby an IKK2SSEE/RelB/p52 signaling axis is sufficient to mediate osteolysis. This proposition is further enforced by the fact that cells lacking both RelB and p52 subunits fail to generate meaningful osteoclasts when transduced with IKK2SSEE. In agreement with this phenomenon, several reports established cross-talk and interdependence of the classical and alternative NF-κB pathways in different responses [20]–[22]. In this regard, it has been shown that TNF, acting through the classical NF-κB pathway, up-regulates RelB expression in human umbilical vein endothelial cells [22]. In our case, we presume that direct activation of the non-canonical effectors, RelB and P52, by IKK2SSEE may represent uncoupling of the pro-inflammatory and pro-osteoclastogenic effects of constitutively active IKK2 in marrow macrophages.

Our study provides evidence for distinct osteoclastogenic events emanating from RANKL signaling and IKK2SSEE. Whereas RANKL induces osteoclastogenesis presumably through activation of multiple coordinated pathways, the IKK2SSEE osteoclastogenic signal is rather restricted to NF-κB-dependent transcription. Although the outcome of both processes appears similar, there is mild morphological difference between RANKL and IKK2SSEE-generated osteoclasts in culture. This variance could be related to differences in cell adhesion, motility, or cytoskeletal arrangement. Nevertheless, despite this change, IKK2SSEE-derived osteoclasts are fully functional in vitro and in vivo. In other studies, it has been shown that whereas IKK1 is required for RANKL-induced osteoclastogenesis, it is dismissible for TNF and IL-1β-induced osteoclastogenesis [12], [23], [24]. In fact, IKK1-null progenitors fail to differentiate into osteoclasts in response to RANKL in vitro yet they form osteoclasts in response to inflammatory stimuli including TNF and IL-1 [12]. Our findings suggest that although RANKL, TNF, and IL1β induce osteoclastogenesis through many signaling pathways, IKK2SSEE appears to do so independently. Thus, multiple osteoclastogenic pathways unique to RANKL, TNF, IL-1β, and IKK2SSEE may exist. The biological significance of each pathway remains obscure and may rely on the unique transcriptional signature each transmits. In the case of IKK2SSEE, the transcriptional complex contains at least RelB and p52 subunits.

In summary, we have identified a unique and novel cross-talk between the canonical and non-canonical NF-κB pathway which appears sufficient to induce osteoclast formation in vitro and bone loss in vivo. This phenomenon is cell-autonomous and independent of RANKL and IKK1. Future studies will attempt to verify the transcriptional signature of this event.

## Materials and Methods

### Reagents

P65, P50 and P52 antibodies were purchased from Cell Signaling Technology (Danvers, MA). Polyclonal anti- RelB antibody was purchased from Santa Cruz Biotechnology Inc (Santa Cruz, CA). Monoclonal anti β-actin and polyclonal anti-Flag antibodies were obtained from Sigma-Aldrich (St. Louis, MO).

### Generation of mice

We used R26StopIkk2SSEE mice in which a cDNA encoding IKK2 containing two serine to glutamate substitutions in the activation loop of the kinase domain, preceded by a loxP-flanked STOP cassette, was cloned into the ubiquitously expressed ROSA26 locus [19]. We crossed mice carrying this allele to the CD11b-cre mice in order to express IKK2SSEE exclusively in myeloid cells (osteoclast progenitors). Both macrophages and osteoclasts derived from CD11bCre/R26StopIKK2SSEE express Flag-tagged IKK2SSEE detectable by Western blot.

### Mouse osteoclasts

Bone marrow macrophages were isolated from long bones of 6- to 8-week-old mice as described previously [11]. Osteoclasts were generated by culturing bone marrow macrophages with RANKL (50 ng/ml) and M-CSF (10 ng/ml) for 4–5 days, or by introduction of pMx-IKK2SSEE retroviral particles into marrow macrophages and culturing with M-CSF for 4–5 days.

### Plasmids and retrovirus generation

IKK2SSEE, P52, RelB were cloned into the pMx retroviral vector fused with either HA or FLAG. To generate retrovirus, PLAT-E cells, which stably express retroviral packaging genes, were transfected with expression vector by using transient transfection reagent. Viral supernatants were collected on day 2 and day 3 post-transfection and immediately used to infect freshly isolated bone marrow macrophages. After 24 h, medium containing the appropriate selection (2 µg/ml puromycin or 1 µg/ml blasticidin) was added to cells for 48 h to select for virus-expressing cells.

### Western blot analysis

Bone marrow macrophages or osteoclasts starved for 6 hours in serum-free medium were stimulated with RANKL (50 ng/ml) and M-CSF (10 ng/ml) and lysed at the times indicated (per experimental conditions) in RIPA lysis buffer supplemented with protease inhibitors and sodium orthovanadate. Equal amount of total cell lysate from each condition was loaded subjected to SDS-PAGE electrophoresis and immunoblots.

### Cytokine measurements

Serum levels of RANKL, OPG, TNF, and IL-1β were measured using commercial ELISA kits (R&D systems, Minneapolis, MN) and following manufacturer's instructions.

### Real-time PCR

Total RNA was isolated from cell cultures at various times using the RNeasy Mini Kit (Qiagen) and was reverse transcribed to cDNAs using SuperScript II according to the manufacturer's instructions (Invitrogen). Primers specific for murine NFATc1, TRAP, GAPDH, cathepsin K, and calcitonin receptor were used. For quantitative real-time PCR, NFATc1 was amplified using 5′-CCCGTCACATTCTGGTCCAT-3′ and 5′-CAAGTAACCGTGTAGCTGCACAA-3′. TRAP was amplified using 5′-CAGCTCCCTAGAAGATGGATTCAT-3′ and 5′-GTCAGGAGTGGGAGCCATATG-3′; cathepsin K using 5′-ATGTGGGTGTTCAAGTTTCTGC-3′ and 5′-CCACAAGATTCTGGGGACTC-3′; and calcitonin receptor using 5′-CAAGAACCTTAGCTGCCAGAG-3′ and 5′-CAAGCACGCGGACAATGTTG-3′; GAPDH using 5′-TGTGTCCGTCGTGGATCTGA-3′and 5′-CCTGCTTCACCACCTTCTTGA -3′. SYBR green dye was used for detection of the product using the SYBR Green PCR Master Mix assay (Applied Biosystems). The standard curve used series of duplicate dilutions of plasmid for each gene and β-actin cDNA. The amplification reaction was performed for 40 cycles with denaturation at 95°C for 10 minutes, followed by annealing at 95°C for 15 seconds and extension and detection at 60°C for 1 minute. The relative RNA abundance of each target gene transcript was normalized against endogenous gene control.

### siRNA knockdown

siRNA oligos were purchased from Thermo Scientific Dharmacon and used to achieve gene knockdown in marrow macrophage cultures. Cells were plated in 6-well tissue culture plates. After one day, cells were transfected with 10 ul of X-treme siRNA transfection reagents (Roche, Indianapolis, IN) and 10 ul of pooled 5 uM siRNA oligos (Thermo Scientific Dharmacon, Lafayette, CO) according to manufacturer's protocol. Cells were further cultured for 5 days and TRAP-stained or lysed for Western blot assays.

### Histology

Intact limbs were preserved in 10% buffered formalin (24 h), skinned, and subjected to a decalcification process using 10% EDTA, pH 7.0, for 7 days with gentle rocking and daily replacement of solution. Decalcified bones were then dehydrated in graded alcohol, cleared through xylene, and embedded in paraffin. Paraffin blocks were sectioned longitudinally. Five-micron sections were then stained with hematoxylin and eosin or histochemically with tartrate-resistant acid phosphatase (TRAP) to determine osteoclasts.

### Micro-Computed Tomography (µCT)

Mouse bones were scanned using microCT (µCT 40, Scanco Medical, Switzerland). The proximal metaphysis regions of tibias were scanned to assess trabecular bone morphology (with the following parameters: 55 kVp, 145 mA, standard resolution, 16.4-mm diameter, 16-mm voxel size, 300-ms integration time). The area of interest was identified just distal to the growth plate and spans a height of 480 mm (30 slices). Following the manufacturer's 3D analysis tools, we measured bone mineral density (BMD), fraction of bone volume known as bone volume over total volume (BV/TV), trabecular thickness (Tb.Th), trabecular separation (Tb.Sp), and trabecular number (Tb.N).

### Statistical analysis

Experiments were repeated at least three times. Changes were assessed using paired t tests. Statistically significant differences were considered at p<0.05. In relevant experiments, we used 6–9 mice per group to achieve statistical significance based on expected effect size of 25%.

All animals are housed in the facility at Washington University under the care of trained technicians and veterinarians. Animals experiencing pain or discomfort following procedures are treated with analgesics per approved protocol. Euthanasia is performed by inhalation of gaseous CO2 in a closed chamber. This method is approved by the AVMA. All animal work was pre-approved by the animal committee of Washington University and conducted according to AVMA guidelines.

## References

[1] Teitelbaum SL, Ross FP (2003). Genetic regulation of osteoclast development and function.. Nat Rev Genet.

[2] Teitelbaum SL (2006). Osteoclasts; culprits in inflammatory osteolysis.. Arthritis Research & Therapy.

[3] Hunter DJ, Sambrook PN (2000). Bone loss. Epidemiology of bone loss.. Arthritis Research.

[4] Khosla S (2001). Minireview: the OPG/RANKL/RANK system.. Endocrinology.

[5] Abu-Amer Y, Darwech I, Otero J (2008). Role of the NF-kappaB axis in immune modulation of osteoclasts and bone loss.. Autoimmunity.

[6] Franzoso G, Carlson L, Poljak L, Shores E, Brown K (1997). Requirment for NF-kB in osteoclast and B-cell development.. Genes & Development.

[7] Baldwin A (2001). The transcription factor NF-κB and human disease.. J Clin Invest.

[8] Tak P, Firestein G (2001). NF-kB: a key role in inflammatory diseases.. J Clin Invest.

[9] Novack DV, Yin L, Hagen-Stapleton A, Schreiber RD, Goeddel DV (2003). The I-kappaB function of NF-kappaB2 p100 controls stimulated osteoclastogenesis.. J Exp Med.

[10] Chaisson ML, Branstetter DG, Derry JM, Armstrong AP, Tometsko ME (2004). Osteoclast differentiation is impaired in the absence of IkB kinase-alpha.. The Journal of Biological Chemistry.

[11] Otero JE, Dai S, Foglia D, Alhawagri M, Vacher J (2008). Defective osteoclastogenesis by IKKbeta-null precursors is a result of receptor activator of NF-kappaB ligand (RANKL)-induced JNK-dependent apoptosis and impaired differentiation.. J Biol Chem.

[12] Ruocco MG, Maeda S, Park JM, Lawrence T, Hsu L-C (2005). IkB kinase-beta, but not IKK-alpha, is a critical mediator of osteoclast survival and is required for inflammation-induced bone loss.. J Exp Med.

[13] Darwech I, Otero J, Alhawagri M, Dai S, Abu-Amer Y (2009). Impediment of NEMO Oligomerization Inhibits Osteoclastogenesis and Osteolysis.. Journal of Cellular Biochemistry.

[14] Abu-Amer Y (2005). Advances in osteoclast differentiation and function.. Curr Drug Targets Immune Endocr Metabol Disord.

[15] Boyce B, Xing L, Fransozo G, Siebenlist U (1999). Required and nonessential functions of nuclear factor-kB in bone cells.. Bone.

[16] Novack DV (2010). Role of NF-kappaB in the skeleton.. Cell Res.

[17] Yao Z, Xing L, Boyce BF (2009). NF-kappaB p100 limits TNF-induced bone resorption in mice by a TRAF3-dependent mechanism.. J Clin Invest.

[18] Otero JE, Dai S, Alhawagri MA, Darwech I, Abu-Amer Y (2010). IKKbeta activation is sufficient for RANK-independent osteoclast differentiation and osteolysis.. Journal of bone and mineral research: the official journal of the American Society for Bone and Mineral Research.

[19] Sasaki Y, Derudder E, Hobeika E, Pelanda R, Reth M (2006). Canonical NF-[kappa]B Activity, Dispensable for B Cell Development, Replaces BAFF-Receptor Signals and Promotes B Cell Proliferation upon Activation.. Immunity.

[20] Kim HB, Evans I, Smallwood R, Holcombe M, Qwarnstrom EE (2010). NIK and IKKβ interdependence in NF-κB signalling-Flux analysis of regulation through metabolites.. BioSystems.

[21] Kim JY, Morgan M, Kim DG, Lee JY, Bai L (2011). TNFα-induced noncanonical NF-κB activation is attenuated by RIP1 through stabilization of TRAF2.. Journal of Cell Science.

[22] Madge LA, May MJ (2010). Classical NF-κB activation negatively regulates noncanonical NF-κB-dependent CXCL12 expression.. Journal of Biological Chemistry.

[23] Ruocco MG, Karin M (2007). Control of osteoclast activity and bone loss by IKK subunits: new targets for therapy.. Advances in Experimental Medicine & Biology.

[24] Boyce BF, Yao Z, Xing L (2010). Functions of nuclear factor kappaB in bone.. Ann N Y Acad Sci.

